# Multiple Copies of Mobile Tigecycline Resistance Efflux Pump Gene Cluster *tmexC2D2.2-toprJ2* Identified in Chromosome of *Aeromonas* spp.

**DOI:** 10.1128/spectrum.03468-22

**Published:** 2022-11-10

**Authors:** Cheng-Zhen Wang, Xun Gao, Jie-Ying Tu, Lu-Chao Lv, Wen-Xian Pu, Xiao-Tong He, Yan-Xiang Jiao, Yu-Ting Deng, Jian-Hua Liu

**Affiliations:** a Guangdong Provincial Key Laboratory of Veterinary Pharmaceutics Development and Safety Evaluation, College of Veterinary Medicine, South China Agricultural University, Guangzhou, China; b Key Laboratory of Zoonosis of Ministry of Agricultural and Rural Affairs, College of Veterinary Medicine, South China Agricultural University, Guangzhou, China; c National Risk Assessment Laboratory for Antimicrobial Resistance of Microorganisms in Animals, College of Veterinary Medicine, South China Agricultural University, Guangzhou, China; d Guangdong Laboratory for Lingnan Modern Agriculture, Guangzhou, China; e Key Laboratory of Fishery Drug Development, Ministry of Agriculture, Pearl River Fisheries Research Institute, Chinese Academy of Fishery Sciences, Guangzhou, China; f Key Laboratory of Aquatic Animal Immune Technology, Pearl River Fisheries Research Institute, Chinese Academy of Fishery Sciences, Guangzhou, China; Yangzhou University

**Keywords:** tigecycline resistance, *Aeromonas*, efflux pump, *tmexC2D2.2-toprJ2*, mobile genetic element

## Abstract

The appearance and prevalence of novel plasmid-encoded tigecycline resistance efflux pump gene clusters *tmexC1D1-toprJ1* and *tmexC2D2-toprJ2* in *Enterobacteriaceae* have raised a threat to public health. Here, another tigecycline resistance gene cluster, *tmexC2D2.2-toprJ2*, was identified in two *Aeromonas* isolates recovered from fish meat and vegetables. Cloning confirmed the expression of *tmexC2D2.2-toprJ2* mediated the resistance to tigecycline and decreased susceptibility to tetracyclines and cephalosporins in both Escherichia coli and *Aeromonas*. In an Aeromonas veronii strain, four copies of *tmexC2D2.2-toprJ2* were located on the chromosome. Further analysis revealed that *tmexC2D2.2-toprJ2* has been detected in the chromosomes of *A. veronii*, Aeromonas hydrophila, and Aeromonas caviae with one to four copies due to the insertion of a potential integrative transferable unit. The occurrence of multiple copies of chromosomal *tmexC2D2.2-toprJ2* may act as a sink for this tigecycline resistance gene cluster, which requires continuous monitoring.

**IMPORTANCE** Tigecycline is regarded as one of the few effective drugs against multidrug-resistant bacterial infection. However, mobile tigecycline resistance efflux pump gene clusters such as *tmexC1D1-toprJ1* and its variants have been identified in both animal- and human-origin *Enterobacteriacea*e. In this study, we first found another efflux pump gene cluster, *tmexC2D2.2-toprJ2*, in the *Aeromonas* chromosome. This gene cluster could mediate tigecycline resistance and decrease susceptibility to tetracyclines and cephalosporins in the *Aeromonas* host strain. Meanwhile, *tmexC2D2.2-toprJ2* was detected with multiple copies in *Aeromonas* spp. This multidrug resistance efflux pump gene cluster with multiple copy numbers might stably exist in *Aeromonas* and serve as a reservoir for *tmexCD2-toprJ2*, facilitating its persistent presence and spread.

## OBSERVATION

Antimicrobial resistance (AMR) is increasingly threatening public health, leaving tigecycline as one of the few effective drugs against multidrug-resistant bacteria (1, 2). The emergence of two types of novel mobile tigecycline resistance mechanisms, including plasmid-encoded efflux pump gene clusters (*tmexCD1-toprJ1*, *tmexCD2-toprJ2*, *tmexCD3-toprJ1b*, and *tmexCD4-toprJ4*) and the tigecycline-modifying enzyme Tet(X4) and its variants, has severely compromised the efficacy of this last-resort antibiotic (3–7). In addition to tigecycline, plasmid-carried *tmexCD-toprJ*-like efflux pump gene clusters can also confer resistance to other antimicrobials (3–6). The *tmexCD-toprJ*-like gene clusters have been identified mainly in China, but have disseminated to other countries (6, 8). *tmexCD1-toprJ1* is the most widely disseminated gene cluster and has been identified in isolates from various sources, with chickens and Klebsiella pneumoniae being the major carrier and host bacterium, respectively (4, 9). In contrast, *tmexCD2-toprJ2* is commonly found in patient-derived samples (6, 10, 11). Recent investigations have found *tmexCD2-toprJ2* to be frequently accompanied by carbapenem resistance genes in *Raoultella* and Klebsiella strains, leading to resistance to both tigecycline and carbapenem (6, 10, 11). Although *tmexCD2-toprJ2* was primarily identified in plasmids of Klebsiella and *Raoultella* spp., whether it has spread and conferred tigecycline resistance in other bacterial species remains unknown. In the present study, we characterized two foodborne *Aeromonas* strains carrying multiply copies of chromosomal *tmexCD2-toprJ2* for the first time.

In December 2021, 45 retail fish meat and 125 fresh vegetable samples were collected from 20 farmers’ markets in Guangzhou, China. Strains GD21SC2284TT and GD21SC2322TT were isolated from fish meat and vegetables, respectively, using MacConkey agar supplemented with 4 mg/L tigecycline. The two isolates were identified as Aeromonas hydrophila and Aeromonas veronii using matrix-assisted laser desorption ionization–time of flight mass spectrometry (MALDI-TOF MS) (Bruker Daltonics, Bremen, Germany). Further PCR and Sanger sequencing confirmed that these two strains were positive for the *tmexCD2-toprJ2* efflux pump gene cluster.

Antimicrobial susceptibility testing profiles of the two *Aeromonas* strains were determined using the broth or agar dilution method recommended by the Clinical and Laboratory Standards Institute (CLSI) with Escherichia coli ATCC 25922 as the quality control strain. The results were interpreted according to CLSI documents (12, 13). Both strains exhibited resistance to tigecycline (8 mg/L), tetracycline (128 mg/L), minocycline, doxycycline, gentamicin, cefquinome, ciprofloxacin (64 mg/L), and florfenicol (Table 1 and see Table S1 in the supplemental material). The two strains were susceptible to ceftazidime and imipenem (both 0.06 mg/L), as well as colistin (2 mg/L). To determine the profile of antibiotic resistance genes (ARGs) and the location of *tmexCD2-toprJ2*, the two *Aeromonas* strains were further subjected to whole-genome sequencing using the Illumina HiSeq and Nanopore MinION platforms. The complete genome data were assembled using Unicycler v.0.4.8 (14). Strain GD21SC2322TT contained a 4.70-Mb chromosome and a plasmid. The strain harbored 10 ARGs, including *bla*_TEM-1B_, *aac(6′)-Ib-cr*, *tet*(A), and *qnrS2* (Table S1), and *tmexCD2-toprJ2* was located on the chromosome. GD21SC2284TT harbored a 5.01-Mb chromosome and two plasmids, wherein chromosomal *tmexCD2-toprJ2* and β-lactamase genes *bla*_CTX-M-3_ and *bla*_TEM-1B_, together with other 14 ARGs, were identified (Table S1).

**TABLE 1:** MICs of various antimicrobial agents against *tmexC2D2.2-toprJ2*-carrying strains and transformants^*a*^

Strain	Strain information	MIC (mg/L) of^*a*^:								
		TIG	MIN	DOX	TET	CTX	CAZ	FEP	CQM	CIP
Aeromonas veronii GD21SC2322TT	Vegetable	8	16	128	128	0.125	0.5	1	2	64
Aeromonas hydrophila GD21SC2284TT	Fish meat	8	32	128	128	16	2	8	>128	64
E. coli										
DH5α	E. coli recipient strain	0.25	1	1	1	0.015	0.06	0.03	0.03	0.001
DH5α/pHSG575	Transformant with low-copy-number vector pHSG575	0.25	1	1	1	0.015	0.06	0.03	0.03	0.001
DH5α/pHSG575-tmexC2D2-toprJ2	Transformants expressing tmexC2-tmexD2-toprJ2	2	4	4	4	0.125	0.25	0.125	0.25	0.008
DH5α/pHSG575-tmexC2D2.2-toprJ2	Transformants expressing tmexC2-tmexD2.2-toprJ2	2	4	4	4	0.125	0.25	0.125	0.25	0.008
DH5α/pHGR	Transformant with medium-copy-number vector pHGR	0.25	1	1	1	0.015	0.06	0.03	0.03	0.001
DH5α/pHGR-tmexC2D2.2-toprJ2	Transformants expressing tmexC2-tmexD2.2-toprJ2	4	8	8	8	0.25	0.5	0.5	1	0.008
A. veroniii										
1AV	A. veroniii recipient strain	0.25	0.5	0.25	0.25	0.015	0.125	0.03	0.06	0.004
1AV/pHGR	A. veroniii strain expressing empty vector	0.25	0.5	0.25	0.25	0.015	0.125	0.03	0.06	0.004
1AV/pHGR-tmexC2D2.2-toprJ2	A. veroniii strain expressing tmexC2-tmexD2.2-toprJ2	4	4	1	1	0.06	0.25	0.25	0.5	0.008

a TIG, tigecycline; MIN, minocycline; DOX, doxycycline; TET, tetracycline; CTX, cefotaxime; CAZ, ceftazidime; FEP, cefepime; CQM, cefquinome; CIP, ciprofloxacin.

**FIG 1:**
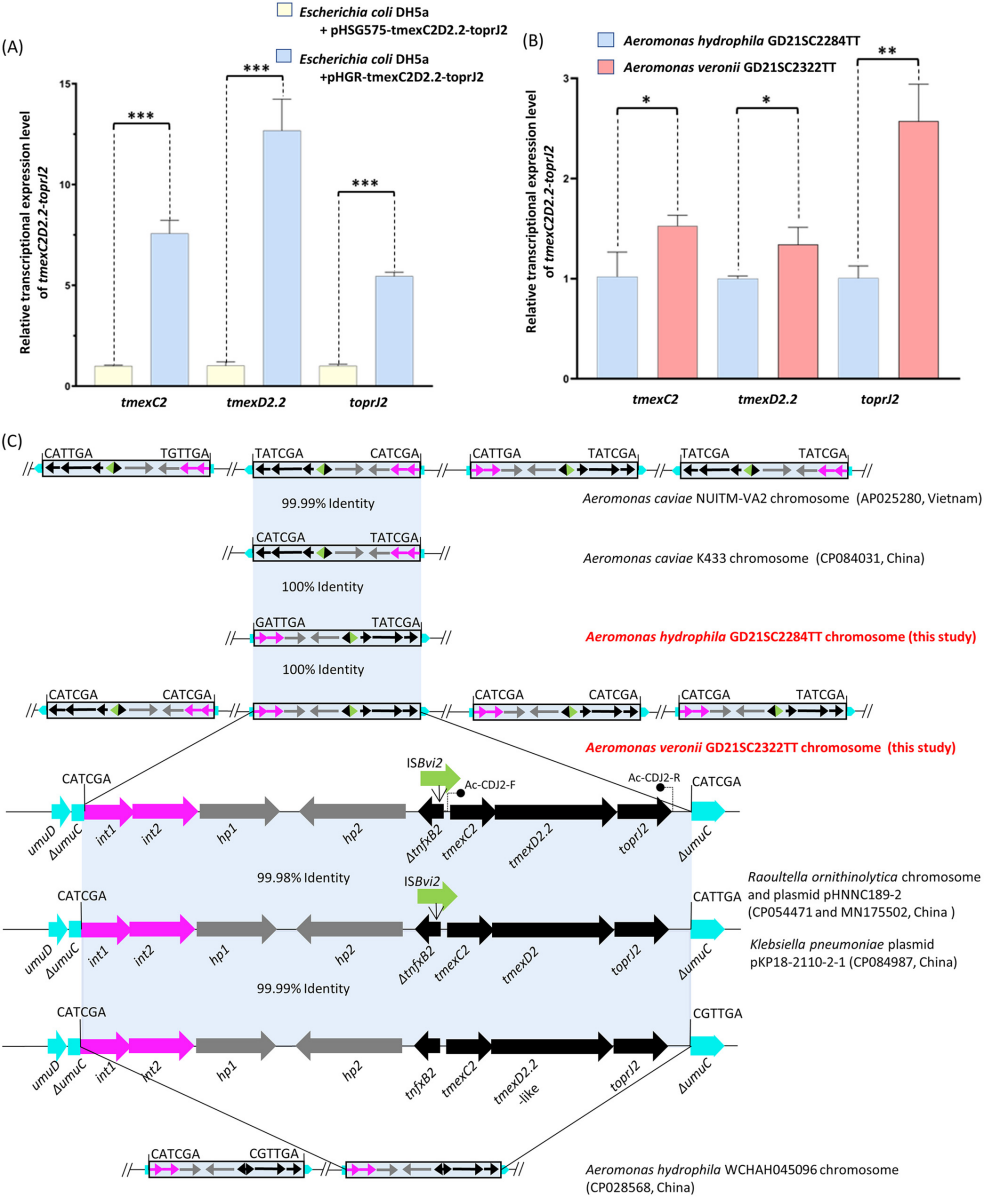
Transcription level of *tmexC2D2.2-toprJ2* in two Escherichia coli recombinant strains (A) and two wild-type *Aeromonas* strains (B). (A) Transcriptional expression level of *tmexC2D2.2-toprJ2* in DH5α carrying pHGR-tmexC2D2.2-toprJ2 compared with that in DH5α carrying pHSG575-tmexC2D2.2-toprJ2; (B) expression level of *tmexC2D2.2-toprJ2* in Aeromonas veronii GD21SC2322TT compared with that of Aeromonas hydrophila GD21SC2284TT. The data were analyzed by quantitative reverse transcription-PCR (qRT-PCR) and are shown as the relative mRNA fold changes normalized to 16S rRNA. Significant differences were analyzed by the unpaired Student's *t* test and are shown as follows: ***, *P* < 0.001; **, *P* < 0.01; *, *P* < 0.05. (C) Comparison of the genetic environment of *tmexC2D2.2-toprJ2* in *Aeromonas* strains with those of closely similar sequences. Arrows with different colors indicate the extents and directions of different genes. The shaded region represents the 99% and 100% homology areas. The *tmexC2D2.2-toprJ2*-bearing IME regions are shown in the box, and the insertion sites are directly indicated. The truncated gene is indicated by the symbol Δ.

The nucleotide sequences of the *tmexCD2-toprJ2* gene cluster identified in the two *Aeromonas* strains were identical. Moreover, compared to the earliest reported *tmexCD2-toprJ2* cluster (6), the efflux pump gene cluster found in this study had one nucleotide difference (T167A) in *tmexD2*, resulting in an amino acid change (Val56Glu) in TMexD2. This gene cluster was designated *tmexC2-tmexD2.2-toprJ2*. To assess the effect of this amino acid difference in TMexD2.2 on the function of TMexCD2-TOprJ2, the DNA fragment of *tmexC2D2.2-toprJ2* was amplified and cloned into the low-copy-number vector pHSG575 with the primers listed in Table S2 and then transformed into E. coli DH5α. Compared with E. coli DH5α carrying empty vector, an 8-fold increase in tigecycline MIC and a 4- to 8-fold increase in MICs of other tetracyclines and cephalosporins were observed in E. coli host strain carrying pHSG575-tmexC2D2.2-toprJ2. Relative to the previously reported antibiotic resistance phenotype mediated by *tmexCD2-toprJ2* (6), the same MIC values against various drugs were observed in the E. coli host strain expressing *tmexC2D2.2-toprJ2*. The findings indicated that this amino acid substitution (Val56Glu) in TMexD2 does not affect the function of the TMexCD2-TOprJ2 efflux system. To further investigate the function of TMexC2D2.2-TOprJ2 in *Aeromonas* spp., *tmexC2D2.2-toprJ2* was cloned into the plasmid pHGR, a broad-host-range vector with medium copy number, and transformed into E. coli DH5α cells. The recombinant plasmid pHGR-tmexC2D2.2-toprJ2 with the correct sequences was further electroporated into environmental *A. veronii* strain 1AV. The MIC values of tigecycline and cephalosporins in E. coli DH5α bearing pHGR-tmexC2D2.2-toprJ2 were 2-fold higher than those of the same host strain harboring pHSG575-tmexC2D2.2-toprJ2 and pHSG575-tmexC2D2-toprJ2, which was due to the 5-fold to 14-fold-higher expression of the *tmexC2D2.2-toprJ2* gene cluster in the pHGR vector than that in pHSG575 (Fig. 1A). Furthermore, the expression of *tmexC2D2.2-toprJ2* in *Aeromonas* host strain 1AV resulted in a 16-fold increase in tigecycline MIC relative to that of the empty vector, indicating that *tmexC2D2.2-toprJ2* could also confer tigecycline resistance in *Aeromonas*. The recombinant strain *A. veronii* 1AV harboring pHGR-tmexC2D2.2-toprJ2 exhibited 2- to 8-fold-higher MICs of other tetracyclines, cephalosporins, and ciprofloxacin than strains carrying empty plasmids. These results revealed that TMexC2D2.2-TOprJ2 could mediate tigecycline resistance and confer decreased susceptibility to multiple drugs in *Aeromonas*.

While GD21SC2284TT contained one copy of *tmexC2D2.2-toprJ2* on the chromosome, intriguingly, four copies of *tmexC2D2.2-toprJ2* were found on the chromosome of GD21SC2322TT, with genetic locations far from each other, two of which were relatively close (distance of 51 kb). Notably, although the same level of tigecycline resistance was observed between the two *tmexC2D2.2-toprJ2*-harboring *Aeromonas* strains (Table 1), GD21SC2322TT exhibited a higher *tmexC2D2.2-toprJ2* transcriptional expression level than that of GD21SC2284TT (Fig. 1B). To find out how the multiply copies of *tmexC2D2.2-toprJ2* appeared in strain GD21SC2322TT, the genetic context of *tmexC2D2.2-toprJ2* was analyzed. Similarly to *tmexCD1-toprJ1*, *tmexCD2-toprJ2*, and *tmexCD3-toprJ1b*, chromosomal *tmexC2D2.2-toprJ2* gene clusters in GD21SC2284TT and GD21SC2322TT were surrounded by the same upstream genetic structures, containing two integrase genes (*int1* and *int2*) and two hypothetical genes (*hp1* and *hp2*) (4–6), which formed an *int1-*like–*int2-*like–*hp1–hp2–tnfxB2*–IS*Bvi2–tmexC2–tmexD2.2–toprJ2* structure. The structure was assumed to be a novel integrative and mobilizable element (IME) (15). Furthermore, similarly to the IME units bearing *tmexCD2-toprJ2* and *tmexCD3-toprJ1b*, the five *tmexC2D2.2-toprJ2* IME units in GD21SC2322TT and GD21SC2284TT were all specifically inserted into *umuC*-like genes. Although the sequences of these *umuC* genes were not completely identical, relatively conserved insertion sites of these IME units were observed (Fig. 1C). BLAST analysis revealed multiple copies of *tmexC2D2.2-toprJ2* in other *Aeromonas* species. Aeromonas caviae strain NUITM-VA2 from Vietnam (AP025280.1) carried four copies of the chromosome-located *tmexC2D2.2-toprJ2*, whereas two copies of *tmexC2D2.2-toprJ2*-like gene cluster, with one nucleotide difference compared with *tmexD2.2*, were found on the chromosome of the environmental A. hydrophila strain WCHAH045096 from China (CP028568.2). A single copy of chromosomal *tmexC2D2.2-toprJ2* was identified in the human-derived *A. caviae* strain K433 from China (CP084031.1). The IME units carrying *tmexC2D2.2-toprJ2* in these three *Aeromonas* strains were also inserted in *umuC-*like genes with similar insertion sites (Fig. 1C). In addition, our previously reported *tmexCD2-toprJ2* IME identified in Raoultella ornithinolytica had two copies, of which one was inserted into the chromosome and another in the plasmid (6), illustrating the high transferability of the *tmexCD-toprJ*-like-carrying IME integrative units.

In addition to the multiple copies of *tmexC2D2.2-toprJ2*, two or more copies of other resistance genes, including *tet*(X) variants and β-lactamase genes, have been increasingly found (16–18).

These events are linked to intracellular transposition or recombination events generated by mobile genetic elements (MGEs) (19). For example, *tet*(X4) was observed in the form of tandem repeats in plasmids mediated by IS*CR2* or IS*26* (16). *bla*_NDM_ and *bla*_KPC_ were also observed for their increased copy numbers in clinical isolates after exposure to novel β-lactams, including cefiderocol and ceftazidime-avibactam (17, 18), indicating that selective pressure from antibiotics could facilitate the occurrence of multiple copy numbers of these AMR determinants (20). Consequently, the increased copy number of *tmexC2D2.2-toprJ2* might be due to multiple insertion events mediated by the integrative unit occurring under drug stress, which needs further investigation. Despite the limited widespread transfer risk of chromosomal *tmexC2D2.2-toprJ2*, the multidrug resistance efflux pump gene cluster with multiple copy numbers might stably exist in *Aeromonas* and serve as a reservoir for *tmexC2D2.2-toprJ2*, facilitating the persistent presence of this novel ARG.

In conclusion, two multidrug-resistant *Aeromonas* strains carrying multiple copies of chromosomal *tmexC2D2.2-toprJ2* were reported for the first time. TMexC2D2.2-TOprJ2 confers tigecycline resistance in both E. coli and *Aeromonas* spp. Given the wide distribution of *Aeromonas* species in aquatic environments and humans, the existence of *tmexC2D2.2-toprJ2* with multiple copy numbers on the *Aeromonas* chromosome may act as a sink for this ARG, thus, requiring continuous monitoring.

### Data availability.

The complete genome data of the *Aeromonas* strains GD21SC2322TT and GD21SC2284TT have been deposited in GenBank under accession no. CP102108, CP102109, CP102110, CP102111, and CP102112, respectively.
