# Supplementary material for: Multiple Copies of Mobile Tigecycline Resistance Efflux Pump Gene Cluster tmexC2D2.2-toprJ2 Identified in Chromosome of Aeromonas spp
Source: Microbiol Spectr. 2022 Nov 10;10(6):e03468-22. doi: 10.1128/spectrum.03468-22 (PMC9769766; doi:10.1128/spectrum.03468-22)
Supplement: Supplemental file 1 — Tables S1 and S2. Download spectrum.03468-22-s0001.pdf, PDF file, 0.09 MB [file spectrum.03468-22-s0001.pdf]

Supplemental Material

Table S1 MICs (mg/L) and antibiotics resistance genes of two isolates GD21SC2322TT and GD21SC2284TT

Strains	GD21SC2322TT	GD21SC2284TT
Species	*Aeromonas veronii*	*Aeromonas hydrophila*
Ampicillin	>128	>128
Imipenem	0.06	0.06
Gentamicin	8	16
Apramycin	32	4
Neomycin	2	64
Florfenicol	32	>128
Colistin	2	2
Fosfomycin	16	16
Antibiotics resistance genes	*ampS, bla*$_{\mathrm{CEPH\text{-}A3}}$, *bla*$_{\mathrm{TEM\text{-}1B}}$, *aac(6')-Ib-cr, dfrA1, tmexC2D2.2-toprJ2, tet*(A), *qnrS2, mph(A), floR, sul1, ARR-3*	*ampH, bla*$_{\mathrm{CTX\text{-}M\text{-}3}}$, *bla*$_{\mathrm{OXA\text{-}21}}$, *bla*$_{\mathrm{TEM\text{-}1B}}$, *aac(6')-Ib-cr, aadA16, aph(3'')-Ib, aph(6)-Id, dfrB7, tmexC2D2.2-toprJ2,tet*(E), *qnrS2,floR, sul1, cphA1, ARR-3, catB3*

Table S2 primers used in this study

Primers	Sequence (5'-3')	Purpose
Ac-CDJ2-F	CAGATGCGGGTATTTTGCCTAG	To construct pHSG575-tmexCD2-toprJ2, and pHSG575-tmexCD2.2-toprJ2
Ac-CDJ2-R	CGTCAGACAGTCTTATTCTCGATCAGAC	
Re-575-CDJ2-F	CTAGGCAAAATACCCGCATCTGCGCTCA CAATTCCACACAACATAC	
Re-575-CDJ2-R	CGAGAATAAGACTGTCTGACGCACTGG CCGTCGTTTTACAAC	
Ac-CDJ2-F	CAGATGCGGGTATTTTGCCTAG	To construct pHGR-tmexC2D2.2-toprJ2
Ac-CDJ2-R	CGTCAGACAGTCTTATTCTCGATCAGAC	
RE- CDJ2-phGR-F	GATCGAGAATAAGACTGTCTGACGTA TTTAACGACCCTGCCCTGAAC	
RE- CDJ2-pHGR-R	CTAGGCAAAATACCCGCATCTGCTT CTCAAATGCCTGAGGCCAG	
16sRNA-F	TGTAGCGGTGAAATGCGTAGA	To measure the transcriptional expression level of *tmexC2D2.2-toprJ2* by qRT-PCR
16sRNA-R	CACCTGAGCGTCAGTCTTCGT	
q-tmexC2-F	CCGTTACGAACCACTGGTGAAG	
q-tmexC2-R	GCAGTTTCGACATTGGCCTG	
q-tmexD2.2-F	CAAGGACTGGTCCGAGCGAG	
q-tmexD2.2-R	GAAACCACCCGAGTTGCCCAG	
q-toprJ2-F	GGTCTGGATGGTGCCTTCGTAG	
q-toprJ2-R	GCTGTCGCTGCCGATCTTC	
